# Pregnancy as a journey of care and connection: Indigenous women’s experiences navigating health systems and community support

**DOI:** 10.3389/fpubh.2026.1839105

**Published:** 2026-06-09

**Authors:** Christina M. Pacheco, Makenna Snyder, Kara Hollins, Kilyn R. Parisien, Angela Scott, Antonio Mirás Neira, Landon Lee, Kinsey Huebener, Yvonnes Chen, Ruth Vaccianna, Ashlyn Potter, Sarah Finocchario-Kessler, Taneisha S. Scheuermann

**Affiliations:** 1 Department of Family Medicine and Community Health, University of Kansas Medical Center, Kansas, KS, United States; 2 Department of Population Health, University of Kansas Medical Center, Kansas, KS, United States; 3 Department of Occupational Therapy Education, University of Kansas Medical Center, Kansas, KS, United States; 4 Department of Indigenous Health, University of North Dakota, Grand Forks, ND, United States; 5 College of Medicine, The University of Oklahoma, Oklahoma, OK, United States; 6 Park Hill High School, Kansas, MO, United States; 7 School of Journalism and Mass Communications, University of Kansas, Lawrence, KS, United States; 8 School of Medicine, University of Kansas Medical Center, Kansas, KS, United States

**Keywords:** American Indian and Alaska Native, community health workers, community-based participatory research, maternal health, pregnancy experiences, qualitative research, social determinants of health

## Abstract

**Introduction:**

Pregnancy and childbirth are opportunities for Indigenous communities to welcome new life, integrate meaningful cultural practices, and tangibly strengthen communal wellbeing. Simultaneously, pregnant American Indian and Alaska Native (AIAN) women must navigate complex healthcare systems while experiencing structural inequities that contribute to elevated risks of complications and poor birth outcomes. This study explored the pregnancy experiences of AIAN women, examining how participants navigated healthcare systems, social supports, and structural challenges.

**Methods:**

We conducted 10 semi-structured interviews with AIAN women to map care journeys and the social and structural contexts shaping them across the perinatal period. Participants were aged 18 years or older, self-identified as AIAN, and had a pregnancy within the past 2 years or were currently pregnant in their third trimester. Guided by AIAN community interest-holders, interviews incorporated a narrative pregnancy-journey mapping approach. We used a community-based participatory research (CBPR)-informed team ethnography approach that incorporated both insider and outsider perspectives.

**Results:**

The investigators identified seven themes through their analysis: cultural beliefs and traditions, prior pregnancy experiences, navigating prenatal healthcare systems, social support networks, social stressors, mental health, and birth and postpartum experiences. Many participants expressed a desire for culturally rooted guidance, including access to traditional healers, doulas, and teachings from their tribal communities. Prenatal care often required coordination across multiple healthcare systems. Family members and partners were consistently identified as central sources of emotional and practical support. While participants navigated cardiometabolic conditions and social stressors, their narratives reflected adaptability, advocacy, and survivance across pregnancy and postpartum.

**Discussion:**

Findings highlight the need for culturally grounded, relationship-based approaches to perinatal care for AIAN women. Participants described a strong desire for greater cultural connection during pregnancy. Strengths-based strategies that integrate cultural practices and expand collaboration with doulas, midwives, and community health workers may improve care experiences and support culturally responsive perinatal care.

## 1 Introduction

Pregnancy and childbirth hold profound cultural, familial, and community significance across many American Indian and Alaskan Native (AIAN) communities in North America (1). Within many Indigenous traditions, birth is understood not only as a biological event but also as a ceremony that welcomes new life into the community and affirms connections between family, ancestors, and future generations (1–4). For many AIAN families, pregnancy represents the continuation of cultural knowledge, kinship networks, and community wellbeing across generations (1, 5). Indigenous perspectives often situate pregnancy within relational frameworks that emphasize connection to family, community, and the next generation (4), highlighting the importance of maternal health as central to the survivance of Indigenous peoples. Survivance is a concept describing Indigenous presence, resilience, and the active continuation of cultural life despite histories of colonial disruption (6–10). AIAN mothers frequently play central roles as caregivers, knowledge keepers, and cultural stewards within families and communities (10, 11). In this way, Indigenous motherhood represents not only caregiving but also sustaining cultural continuity and community leadership even as they navigate broader social and structural challenges (10–12).

Despite these strengths, AIAN communities continue to face persistent inequities in maternal and fetal health outcomes in the United States (13). Many high-income countries have experienced declines in maternal mortality over recent decades. In the United States, the reported maternal mortality rates were 18.6 maternal deaths per 100,000 live births in 2023 and 17.9 in 2024 (the decline did not reach statistical significance) (14). These rates remain more than double those observed in peer nations (15). Similar disparities in Indigenous maternal health have also been documented in other settler-colonial contexts, including Canada (16), Australia (17), and New Zealand (18), underscoring the broader importance of culturally grounded and relational approaches to perinatal care among Indigenous populations globally. Within the United States, data from 2019–2023 indicate a maternal mortality rate of 60.8 deaths per 100,000 live births among AIAN women, compared to 19.5 among white women (19). Regional analyses demonstrate substantial variation, with rates among AIAN women in the Midwest reaching 94.2 deaths per 100,000 live births in 2019 (20). In addition to elevated mortality, AIAN women experience a disproportionate burden of pregnancy-related cardiometabolic conditions. In 2022, the rate of gestational diabetes among AIAN was substantially higher among AIAN women (127 per 1,000) compared to white, non-Hispanic women (73 per 1,000) (21). Obesity affects nearly half of all AIAN women and contributes to increased risk of hypertensive disorders of pregnancy, cardiomyopathy, and long-term cardiovascular disease (22, 23).

Clinical risk factors alone may not fully explain these disparities. Structural determinants, like healthcare access, continuity of care, and culturally responsive services, play an important role in shaping pregnancy experiences and outcomes (24). Many AIAN individuals access or initiate care through the Indian Health Service (IHS), a federally funded healthcare system established to fulfill treaty obligations to tribal nations. However, many IHS and tribal facilities lack comprehensive obstetric services, requiring referrals to external hospitals or specialty providers for prenatal care or delivery (25). These transitions across healthcare systems can create fragmented care experiences and challenges in coordinating services during pregnancy (25).

Geographic access to obstetric services further shapes maternal healthcare experiences in many rural and reservation-serving regions (26). Research examining birth records in Montana found that American Indian women traveled significantly farther than white women to deliver and were substantially more likely to give birth at hospitals without obstetric services, reflecting persistent inequities in maternity care infrastructure in rural areas (27). Limited access to specialty obstetric services may also contribute to delays in prenatal care initiation and reduced access to high-risk maternal care (26).

Studies examining prenatal care access among AIAN women have also documented disparities in early engagement with prenatal services. Analysis of Pregnancy Risk Assessment Monitoring System (PRAMS) data in North Dakota found that AIAN women had nearly twice the odds of beginning prenatal care after the first trimester compared to white women and reported a higher prevalence of barriers, including transportation challenges and limited appointment availability (28). These findings emphasize the importance of understanding how structural conditions such as geography, healthcare infrastructure, and resource availability affect access to prenatal care.

Historical and ongoing structural inequities further influence healthcare experiences during pregnancy. Policies such as involuntary sterilization, boarding schools, and broader colonial disruptions to AIAN family and community structures have contributed to intergenerational mistrust of medical institutions and shaped reproductive health experiences for many AIAN women (6, 29). At the same time, AIAN communities continue to demonstrate survivance, reclaiming culturally grounded approaches to pregnancy, birth, and parenting (1–3, 30). Across many communities, efforts to revitalize traditional midwifery, birth ceremonies, and community-based models of care represent forms of Indigenous survivance (8), affirming cultural knowledge, community authority, and self-determination in maternal health (6, 10, 31, 32).

Although prior studies have documented disparities in maternal outcomes and barriers to prenatal care among AIAN women (25, 27, 28), less research has centered Indigenous women’s narratives of pregnancy as relational, culturally grounded, and shaped by interactions across healthcare, family, and community systems. Additionally, qualitative studies focused on AIAN women from the Great Plains region remain limited. This study is unique in its use of a CBPR-informed, narrative journey-mapping approach to examine pregnancy experiences among AIAN women across the prenatal, delivery, and postpartum periods, focusing on how they navigated healthcare systems, social supports, and structural barriers. This work centers Indigenous women’s voices, survivance, cultural teachings, and relational experiences across pregnancy and postpartum. These findings inform the development of a culturally grounded, community-based intervention, SAFE (Securing AIAN Futures through eHealth), which is being designed to address the structural and relational gaps identified by AIAN women. By centering AIAN voices and lived experiences, this work highlights opportunities to strengthen maternal healthcare systems and advance culturally responsive, equity-focused models of care.

## 2 Methods

This was a qualitative study using semi-structured interviews to explore the pregnancy experiences of AIAN women from the Plains region (33, 34). This study was guided by Indigenous scholarship and principles of community-engaged research. The research team includes AIAN and allied researchers and students with long-standing partnerships with Native-serving organizations. Interest-holders (35) contributed to participant recruitment, instrument development, data analysis, interpretation of findings, and manuscript development. These perspectives shaped the development of the study, the interpretation of participants’ narratives, and the commitment to prioritize AIAN voices and lived experiences throughout the research process.

This investigator-initiated study was funded by departmental research funds and approved by the University of Kansas Medical Center Institutional Review Board (STUDY00149826). Participants provided informed consent electronically via the REDCap survey platform and verbally at the start of the interview.

### 2.1 Participant recruitment and eligibility

Recruitment was conducted using a combination of purposive and snowball sampling to identify AIAN women from the Plains region, in partnership with the Kansas City Indian Center. Potential participants were recruited through word-of-mouth outreach, social media posts, and the distribution of flyers and posters in locations frequently visited by AIAN individuals, including partner organizations and community events. To determine eligibility, potential participants completed a 23-item screening survey collecting basic demographic information and pregnancy-related details. Participants qualified if they were 18 years or older, self-identified as American Indian or Alaska Native, and had a pregnancy within the past 2 years or were currently in their third trimester. The gestational age criterion was used to ensure that currently pregnant participants had sufficient interaction with healthcare providers to reflect thoughtfully on their prenatal care experiences. We gathered additional information on healthcare access, insurance coverage, living arrangements and household composition, financial stressors, education, employment status, digital connectivity (e.g., phone and internet access), health information preferences, use of pregnancy-related services (e.g., Special Supplemental Nutrition Program for Women, Infants, and Children (WIC) and community health workers, etc.), and reproductive history.

### 2.2 Interview procedures

#### 2.2.1 Instrument development

Interview guides were developed to elicit detailed narratives of AIAN women’s pregnancy experiences, conceptualized as unfolding over time and shaped by care experiences, decision-making, and social context. Drawing on life course perspectives (36, 37), interviews focused on timing and sequencing of key events, including pregnancy recognition, entry into prenatal care, transitions between providers or healthcare systems, and experiences during delivery and the postpartum period.

The study team developed the interview guide in collaboration with community members and interest-holders at the Kansas City Indian Center to ensure cultural relevance and appropriateness. The feedback informed revisions to the language and content of both survey and interview tools. Interviews were semi-structured and participant-centered, allowing individuals to share their experiences in narrative form. Questions explored interactions with healthcare systems, navigating referrals and specialty care, and sources of support, including family, community resources, cultural knowledge, and practices related to pregnancy and childbirth. Participants were also asked about barriers and facilitators to accessing care, the availability or lack of support (services and interpersonal), and interactions with community health workers (CHWs), doulas, midwives, and other support providers. The full semi-structured interview guide is available in Supplementary file 1.

A narrative journey-mapping approach (38), was used to prompt participants to describe key experiences across pregnancy and postpartum periods and reflect on how these unfolded over time. The interview guide was refined iteratively throughout data collection; following early interviews, additional probes related to pain, urinary incontinence, and sexually transmitted infection screening were added to better capture emerging health concerns.

#### 2.2.2 Data collection

Participants provided informed consent both electronically and verbally prior to the interview. Interviews were conducted between April and December 2023 via Zoom or by telephone, at the participant’s preference. Each interview lasted approximately 45 to 60 min and was conducted by CP, with YC and KP assisting in several interviews. All interviews were audio-recorded and transcribed verbatim using Rev. transcription services. The transcripts were then de-identified, and references to specific tribes, reservations, or geographic locations were removed to protect participant and tribal confidentiality. Participants received a $100 gift card as compensation for their time.

### 2.3 Data analysis

#### 2.3.1 Quantitative data

Screening and demographic survey data were exported as comma-separated values (.csv) files and analyzed using SPSS version 29.0.1.0 (IBM Corp.). Descriptive statistics, including frequencies, percentages, means, and standard deviations, were calculated to characterize the study sample.

#### 2.3.2 Qualitative data

Qualitative analysis was conducted using a community-based participatory research (CBPR)-informed team ethnography approach (39), incorporating both insider and outsider perspectives to deepen understanding of participants’ experiences. Insider perspectives from researchers and collaborators with lived experience or community connections provided culturally grounded interpretation, while outsider perspectives supported the identification of broader patterns and themes within the data.

The research team collaboratively developed a codebook and conducted consensus-based coding using Dedoose Version 9.2.12 (2024) (40). Coding was iterative, with team members meeting regularly to discuss emerging themes, refine codes, and resolve discrepancies. Analytic memos and illustrative excerpts were documented throughout the coding process to support reflexive analysis and identify potential gaps in the data. Themes were developed inductively through iterative review of participant narratives and refined through team consensus discussions informed by CBPR principles and Indigenous-informed interpretation.

### 2.4 Figure development

**Figure 1:**
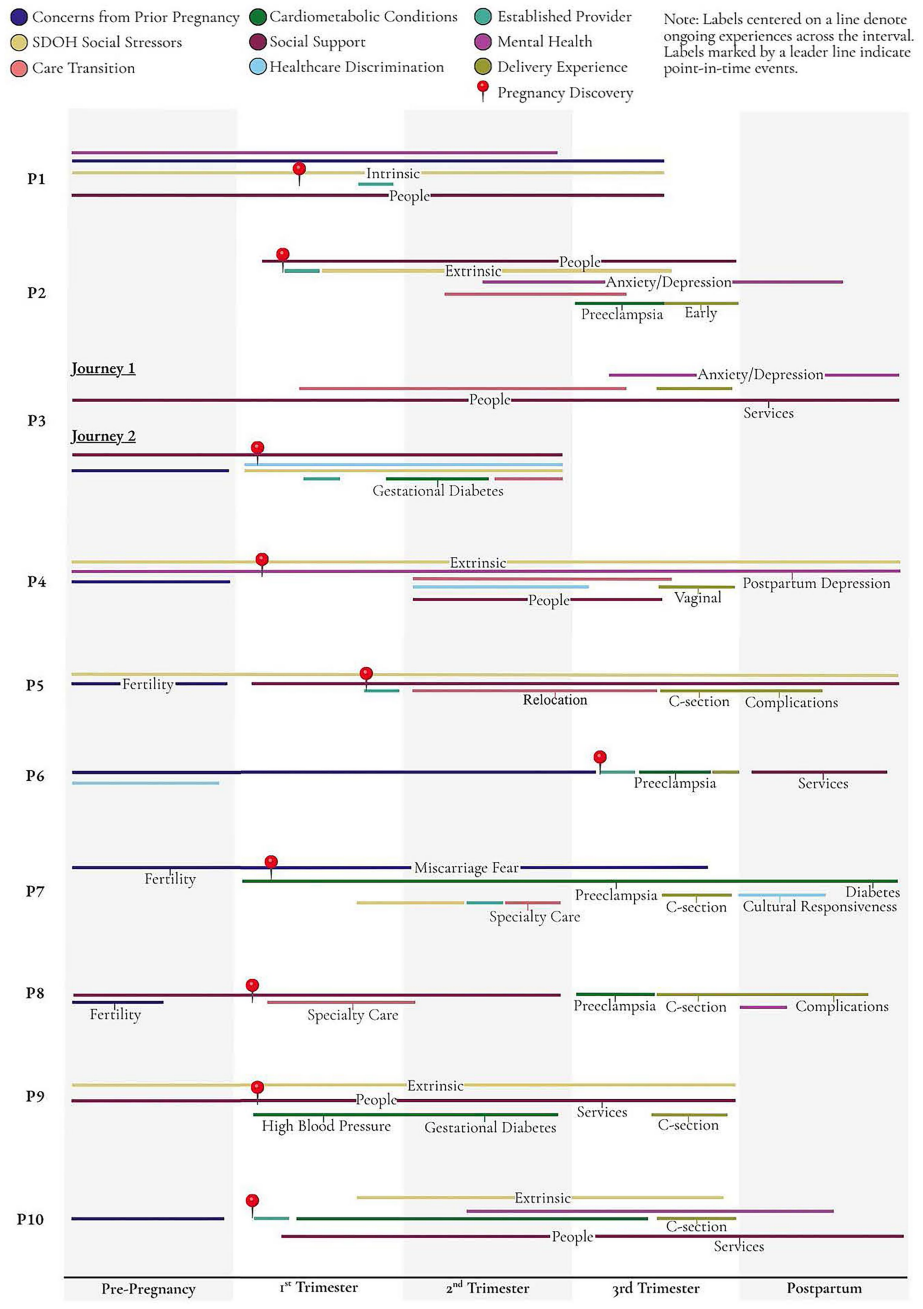
This illustrates each participant’s pregnancy journey, mapping key themes identified in interviews across the perinatal timeline. Although most participants discovered their pregnancy during the first trimester, the timing of initial engagement with prenatal care varied. Participants navigated a range of supports and challenges, including prior pregnancy experiences, social and structural stressors, and transitions across healthcare systems, which shaped their information needs, care-seeking behaviors, and delivery experiences. Mapping these experiences chronologically highlights how clinical events, social stressors, and healthcare interactions often overlapped, influencing care decisions and perceptions of support across pregnancy and into the postpartum period.

After conducting the interviews, the research team synthesized participants’ stories to create visual maps of their pregnancy journeys. These maps showed key moments, including pregnancy recognition, trimesters, and the postpartum period. They helped identify patterns in how participants accessed healthcare and social support, as well as the structural challenges they faced. The aggregated visualization of these mapped experiences is presented in Figure 1, illustrating the timing and co-occurrence of key themes across participants’ pregnancy journeys.

### 2.5 Participant validation and community review

To enhance credibility and ensure respectful representation, preliminary results were shared with participants for feedback on accuracy, interpretation, and potential identification concerns. Participant feedback informed minor revisions to the language and presentation of findings. In addition, the near-final manuscript was shared with the study’s Community Advisory Board (CAB) prior to submission. CAB members provided feedback on the framing and interpretation of findings to ensure alignment with community perspectives and priorities.

## 3 Results

Of the 41 individuals who met the eligibility criteria, 10 completed interviews (24.4%). Reasons for non-participation included being unreachable after three attempts (41.9%), no-shows (12.9%), and unknown reasons (45.2%). Table 1 shows the demographic characteristics of the participants.

**Table 1:** Demographic characteristics of interview participants (*N* = 10).

Variables	Frequency, *n* (%)
Age (median, IQR)	34 (IQR 28–38)
Pregnancy status (at time of interview)	
Postpartum	6 (60)
3rd trimester	2 (20)
2nd trimester**	2 (20)
1st trimester	0 (0)
Race and ethnicity*	
American Indian/Alaska native	10 (100)
Additional race/ethnicities selected	
Black or African American	1 (10)
Multiracial	1 (10)
Latino or Hispanic	2 (20)
Early life geographic setting*	
Reservation or tribal trust land	5 (50)
Rural off-reservation area	1 (10)
Urban or suburban areas	6 (60)
Education status	
Graduate school	4 (40)
College	3 (30)
Some college or tech school	1 (10)
High school or less	2 (20)
Employment status	
Employed full-time	7 (70)
Employed part-time	1 (10)
Student	2 (20)
Insurance*	
Medicaid	6 (60)
Private	4 (40)
Other	2 (20)
Prenatal care services*	
Private/hospital	9 (90)
IHS	4 (40)
Trimester of first prenatal visit	
1st trimester	9 (90)
3rd trimester	1 (10)
Cardiometabolic conditions	
No cardiometabolic conditions	4 (40)
Cardiometabolic conditions	6 (60)
Types of cardiometabolic conditions	
High blood pressure	1 (10)
Gestational diabetes	1 (10)
Pre-eclampsia	2 (20)
High blood pressure and gestational diabetes	1 (10)
Pre-eclampsia and gestational diabetes	1 (10)

*Select all that apply, numbers may not add to 100%; **These participants were interviewed during the second trimester of their current pregnancy but also had previously given birth within the last 2 years before the interview.

All participants identified as AIAN women and were enrolled members of federally recognized tribes. Four participants were pregnant at the time of the interview, and six were postpartum. Participants described diverse upbringing environments, including growing up on a reservation or tribal trust land (*n* = 5), in a rural off-reservation area (*n* = 1), and in urban or suburban areas (*n* = 8). Most participants had completed college or graduate education (*n* = 7) and were employed or enrolled full-time as students (*n* = 9).

Participants often depended on Medicaid for insurance or received healthcare through the Indian Health Service (IHS). Most initiated prenatal care in the first trimester. Nine participants received care at private hospitals or clinics, and four also used IHS services. Financial stress was common; eight participants reported stress related to food or financial resources during pregnancy, and six utilized the WIC program.

Thematic analysis identified seven interconnected themes describing AIAN women’s pregnancy journeys, highlighting both the cultural strengths and structural challenges: (1) cultural beliefs and traditions surrounding pregnancy, (2) prior pregnancy experiences, (3) navigating prenatal healthcare systems, (4) social support networks, (5) social stressors influencing pregnancy experiences, (6) mental health, and (7) birth and postpartum experiences. Figure 1 illustrates how these experiences unfold through pregnancy.

### 3.1 Cultural beliefs and traditions

Participants described pregnancy as extending beyond clinical care to include cultural teachings, family relationships, and intergenerational traditional knowledge. Access to these teachings was often inconsistent, shaped by geography, family disruption, and limited early exposure to traditional practices.

Although no participants reported formally consulting a traditional healer during pregnancy, many expressed a desire for culturally rooted guidance, including access to traditional healers, doulas, or teachings from their own tribal communities. In the absence of these resources, family members were a primary source of cultural knowledge, offering guidance on practices during pregnancy.

Participants described the sharing of intergenerational knowledge among female family members, *“[T]hey helped me, they’d tell me stuff… female family like that, different generations, like my cousins that had kids before me.”* (P3) One participant described how living far from her tribal communities limited her access to cultural knowledge, “*I just mostly got teachings from my mom because we were off the reservation… it would’ve been very different if we had those cultural aspects though, to incorporate into [my] pregnancy.*” (P1).

Teachings were often specific to tribal or family traditions and included not entering the powwow circle while pregnant (“*you can still go, you just can’t go out into the circle;*” P3), keeping hair down during pregnancy because “*a lot of the power that comes from giving birth comes from our hair*” (P1), and avoiding funerals “*because of the spirits that tend to be among them*” (P10). Participants also described teachings about not purchasing baby items too early, noting that “*…in our ways we believe that something could happen*” (P6).

Postpartum teachings include cradleboards, postpartum customs, and practices for introducing the baby to the community. One participant shared, “*You’re not supposed to wrap up the cradle board if there’s not a baby in it…unwrapping it is allowing the spirit to find its home in the cradleboard*” *(P1).* Another described a tradition in which the baby is later presented to the community, “*your baby and your family are coming out into the world and being accepted*” (P9).

#### 3.1.1 Desire for culturally rooted care

Within the healthcare system, participants reported that culture was rarely discussed during prenatal care visits. When asked whether culture came up with medical providers, one participant responded simply, “*No, not at all*” (P4). Others described accommodations they received in their care, if requested. For example, one participant was able to keep her placenta for cultural purposes, noting she asked her provider, “*‘How do we go about that? What does that look like?’ And they’re like, ‘Yeah. You’re able to’*” (P6).

Participants had suggestions for culturally grounded models of care. One described an ideal hospital environment with dedicated space for traditional practices, “*a section in the hospital where you can burn sage, burn cedar, and have a medicine man come in… a very private room where they can do their ceremonies while also being monitored by their doctor… just having a separate area used for traditional healers would be really nice to have*” (P1).

Some participants described more affirming postpartum experiences in Native-serving settings compared to non-Native care settings, citing more coordinated, culturally grounded supports such as lactation specialists, breastfeeding education, and group-based programming. One participant described how these services created opportunities for connection, for “*women to get together and breastfeed together*,” and emphasized that being “*around my people*” increased her comfort and confidence during the postpartum period (P5). The same participant expressed the importance of culturally relevant information for care decisions, such as in the case of circumcision, “*I regret it… But I didn’t have culturally relevant information for a Native male.*”

### 3.2 Prior pregnancy experiences

#### 3.2.1 Experiences of pregnancy complications, fertility concerns, and loss

Participants described prior complications, fertility concerns, and pregnancy loss as especially influential in shaping subsequent pregnancies. These experiences heightened vigilance and affected how participants interpreted bodily changes and engaged with providers. One participant described how a prior placental complication and pregnancy loss shaped her response when providers informed her late in pregnancy that she might have a low-lying placenta, “*They said they found that out during my 20-week anatomy scan, and I’m already in week 28… ‘How are you just barely telling me this now?’… with my first pregnancy, my placenta actually erupted, and we lost her at 25 weeks.*” (P1).

Others described recurrent fears of miscarriage in their subsequent pregnancies. One participant explained that fear remained “*always in the back of my mind,*” after multiple miscarriages, even during pregnancies that progressed further (P7). Fertility concerns also shaped how some participants understood pregnancy. One participant noted that after years of irregular periods, surgery, and possible PCOS, she had come to believe pregnancy “*wasn’t in the cards*” before later discovering she was pregnant (P6). Another participant described IVF as shaping the emotional rhythm of pregnancy from the outset, explaining that she and her spouse knew exactly when their embryo transfer occurred and lived with the uncertainty of “*hopefully we’re still pregnant today*” (P8).

#### 3.2.2 Learning from prior pregnancy care experiences

Participants described how prior pregnancy and prenatal care experiences shaped their expectations, preferences, and decision-making in subsequent pregnancies. One participant contrasted the support she received from a midwife in an earlier pregnancy with more rushed care interactions in a later pregnancy, explaining that with midwifery care felt “*more centered on you rather than, we have 100 more women outside who need to be seen*” (P1).

Others described how previous experiences influenced their perspectives on medical interventions. One participant explained that after a difficult prior experience, she approached later prenatal procedures more cautiously, “*This time I don’t know if I’ll agree to the [Group B Strep] swab or if I’ll ask to self-administer… I think that sometimes doctors want to intervene a little more than is necessary*” (P8). Prior negative outcomes also contributed to a heightened sense of self-advocacy in care settings. As one participant reflected, “*Our negative outcomes have just put me in a different place where it’s like, I know I have to be my own advocate because nobody else is going to be*” (P8).

#### 3.2.3 Changing life circumstances across pregnancies

Participants described how pregnancies unfolded within changing social and family circumstances. These included childcare, employment, school, caregiving, and financial stability. One participant explained that a subsequent pregnancy raised practical concerns because she had only recently secured childcare for her first child, “*We had literally just got my daughter into daycare… and then it was like, ‘Well, we have a second child to worry about now*’” (P3).

Another participant described how her subsequent pregnancy unfolded alongside a new job and ongoing caregiving for her parent, creating “*a lot of different questions and a lot of unknowns*” (P1). Others described balancing pregnancy with school, unstable partner support, or the responsibilities of everyday life in ways that made each pregnancy feel distinct from the last (P2; P4).

### 3.3 Navigating prenatal healthcare systems

Participants described navigating prenatal care across multiple healthcare systems, including tribal clinics, IHS, private hospitals, and specialty providers. Experiences often involved confirming pregnancy through local clinics, coordinating referrals for obstetric services, managing pregnancy-related health conditions, and navigating broader concerns about trust, discrimination, and historical experiences within healthcare systems.

#### 3.3.1 Care transitions, IHS referrals, and accessing specialty care

For many participants, tribal clinics or IHS served as the initial point of contact to confirm pregnancy and initiate prenatal care. Early visits focused on pregnancy confirmation, scheduling follow-up appointments, and identifying available resources. As one participant shared, these visits helped her begin organizing care and accessing support, “*setting up appointments and getting all the information I needed… asking for every available resource*” (P9).

Prenatal care often required coordination across multiple healthcare systems. Because many tribal and IHS clinics do not provide full obstetric services, participants were frequently referred to external hospitals or specialty providers for prenatal care and delivery. One participant described this process, “*I contacted our local clinic… it’s a tribal clinic to get referred out to an OB. So, I went there, they confirmed the pregnancy, and then they referred me out to an OB*” (P10).

These referrals often introduced logistical challenges. Participants described traveling long distances for specialty care and relying on family members or tribal transportation services. One participant explained, “*I only had two [visits] in [my tribal community], and then I got referred out to another hospital… then I got referred to a different place in [a different city]*” (P2). Coordinating care across systems also required navigating insurance and sharing information between clinics, which participants described as complicated, “*It was a learning curve… having to tell two different hospitals or clinics all the information between each other*” (P9).

In some cases, referral processes and scheduling constraints contributed to delays in care. One participant described difficulty securing timely appointments due to limited availability, “*That’s why it took a little bit longer than normal [for my first prenatal visit] because I just didn’t have a whole day to block off at whatever random time I was given*” (P3). Another described confusion when transitioning from fertility care to obstetric care, noting misaligned expectations across providers, “*Our IVF (in vitro fertilization) clinic didn’t release us until week 11… so I didn’t think I had to find somebody sooner. But [the prenatal care provider] expected me to come in before the 12-week mark and were like, ‘Why did you wait so long?’*” (P8).

Despite these challenges, some participants described positive experiences within AIAN-serving care environments. One participant explained that returning to an AIAN-run clinic for postpartum care felt more comfortable because “*it’s all Native women that work there*” (P5). She went on to share how being “*around my people and them understand[ing]*” created a stronger sense of support during her care. (P5).

#### 3.3.2 Monitoring of cardiometabolic and pregnancy-related conditions

Six participants reported cardiometabolic conditions during pregnancy, including preeclampsia (*n* = 3, one also with gestational diabetes), gestational diabetes (*n* = 3, one also with elevated blood pressure), and elevated blood pressure alone (*n* = 1). These conditions often require increased monitoring and changes in care. Participants described both early identification and later-emerging complications.

One participant explained that abnormal laboratory results flagged through IHS led to an earlier-than-expected gestational diabetes diagnosis, “*If IHS didn’t flag that… doctors usually wait until 20 or 24 weeks to test for that, and I got tested within the first three months*” (P3). For one participant, providers increased monitoring after identifying elevated blood pressure and abnormal glucose levels, “*I do daily blood checks and sugar checks, and I do weekly appointments now… blood pressure checks, blood withdrawal checks, and imaging checks*” (P9). Similarly, another participant described transitioning to weekly visits after a late pregnancy diagnosis of high blood pressure, “*When I first found out I had high blood pressure, I was about 30 weeks… they told me I would have to come in every week until I had my baby*” (P2). In another case, an otherwise uncomplicated pregnancy changed rapidly late in gestation, “*We had a completely normal pregnancy until I was due, and then I developed severe pre-eclampsia*” (P8).

#### 3.3.3 Discrimination, mistrust, and historical trauma in care

Participants navigated prenatal care within broader contexts of discrimination, mistrust, and historical trauma. Even without direct experiences of discrimination during pregnancy, many described a heightened awareness of inequities. One participant noted, “*the chances of poor outcomes is just higher for me… that was definitely on my radar*” (P8). She also recalled discomfort when hospital staff focused heavily on her Native identity, “*She was a little weirdly obsessed with the fact that I was a Native person… in a hospital setting, I don’t really want someone to fixate that closely on my race*” (P8).

These concerns sometimes influenced care-seeking decisions. One participant chose to travel farther for delivery after hearing from others that a closer hospital was “*subpar*” and “*didn’t treat the Native population very well*” (P10). Others described more direct experiences of feeling unsupported. One participant explained, “*I tell them I’m Native American, and some of them weren’t supportive, some of the nurses and doctors*” (P4). She added that these experiences made it harder to seek care and required her to advocate “*for myself and on my unborn baby’s behalf*” (P4).

Participants also connected present-day care to historical violence against Native communities. One participant described fears rooted in stories of forced sterilization and children being taken from families shaped her thinking during pregnancy: “*Those are the nightmares I hear about that happened back home*” (P5). She further explained that these histories influenced what she expected might happen in care, including fear that providers might tell her she needed sterilization, “*I thought they were only going to let me get pregnant this many times… I thought they were going to tell me I need to get my tubes tied*” (P5).

### 3.4 Social support networks

#### 3.4.1 Family and relational support

Family members and partners were the most consistent sources of support during pregnancy, providing reassurance, childcare, advice, and help with daily responsibilities. One participant described how her husband and mother supported her physically and emotionally, “*My husband was taking on extra duties in the household with our toddler, the housework, cooking dinner, cleaning the apartment, and letting me rest when I needed to*” (P10). Support also came from partners, friends, and visiting relatives. One participant explained, “*I have my fiancé here. He’s really good, and he’s helpful and supportive as well. And I have family that does come and visit from out of state, so they come and help when they’re here*” (P9).

Female relatives were particularly important sources of guidance. Participants described receiving advice and emotional support from mothers, sisters, and extended kin networks. One participant noted that her mother provided “*verbal support above all else*” (P4), while another relied on her sister after the deaths of both parents (P7). Others highlighted the role of broader intergenerational networks, including cousins, aunties, and grandmothers, in sharing experiential knowledge and support (P5).

#### 3.4.2 Community-based support and paraprofessional roles

In addition to family networks, participants described the value of community-based supports, including community health workers (CHWs), doulas, midwives, case managers, and home-visiting programs. These roles were seen as important for information sharing, advocacy, and more personalized support during pregnancy. One participant contrasted midwifery care with more clinical settings, noting that “*the level of communication is more centered on you*” (P1). Another described how a CHW could fill gaps in care, explaining that “*so much of the information about birth, I’ve had to search out on my own… You have a very limited window of when you get to see [your provider]. If I had other folks I could talk to, that’d be helpful*” (P8).

Participants described direct experiences with these supports, including working with a case manager through the local Indian Center (P4), and receiving transportation assistance from an IHS community health representative (P5). Home-visiting programs were similarly valued. One participant described Healthy Families as providing regular check-ins and resources, noting that “*they’ll have a home worker come once a week… and provide different community resources,*” while also supporting her in managing her gestational diabetes (P3).

#### 3.4.3 Desire for expanded support

Participants described gaps in support, including the need for more culturally grounded care, stronger peer networks, and greater assistance navigating services and decisions. Some participants emphasized feelings of isolation, particularly when living away from community. One participant reflected that a CHW could have helped her connect to resources and other Native women, explaining, “*It was very isolating… I didn’t know anybody here… a CHW could have helped me find resources or support systems for Native women*” (P1). Another described wanting a mother’s group specifically for AIAN women (P4).

Participants expressed interest in doulas or AIAN birth workers who understood their cultural values. One participant explained, “*someone who looks like you and understands your values and culture… that would be such a huge shift of the whole process*” (P1). Others emphasized the importance of advocacy during care encounters, describing a need for “*somebody who’s in my corner*” (P8) and support in asking questions and processing information, “*I didn’t even know what to ask because I didn’t know what I didn’t know*” (P6).

Support needs also included practical information. One participant described wanting more support after a gestational diabetes diagnosis, explaining that it felt like, “*Here, you have this. That’s all we’re giving you*” (P3). Another described confusion around maternity leave and uncertainty about where to seek help (P6).

### 3.5 Social stressors that influence pregnancy experiences

While participants often described drawing on personal resilience and family support, pregnancy frequently occurred alongside other complex life circumstances.

#### 3.5.1 Family and role-related stressors

Participants described pregnancy as involving uncertainty, emotional strain, and the challenge of balancing competing responsibilities. Several participants highlighted the difficulty of managing multiple roles simultaneously, including caring for young children, starting new jobs, or continuing education. One participant described the stress of managing “*two under two*” after only recently arranging daycare for her first child (P3), while another navigated pregnancy alongside a new job, saying she had “*a million different questions*” because of everything happening at once (P1). Others expressed concerns about how pregnancy would affect school, independence, and daily life, particularly when lacking partner support (P2). Caregiving responsibilities added further complexity, with one participant serving as the primary caregiver for her parent with dementia while receiving limited support from siblings (P1).

Intimate partner dynamics were also significant. While some participants described supportive relationships, others described conflict, absence, or violence. One participant described seeking care while experiencing domestic violence and feeling dismissed by healthcare providers when raising concerns, “*I kept on telling them… ‘Yes, I feel this way,’ and they would dismiss me… they wouldn’t talk to me further, and they would just dismiss my feelings*” (P4). Another participant described relocating during pregnancy due to a lack of partner support, moving to create a safer and more stable environment for her child (P5).

Participants also described challenges related to emotional expression during pregnancy. One participant explained that despite encouragement from others, she felt pressure to remain positive and “*should be happy,*” rather than being able to process more difficult emotions. She described feeling “*not allowed to be emotional*” and emphasized the need for space to experience a full range of feelings instead of “*mask[ing] them to be positive the entire time*” (P10).

Pregnancy was also shaped by grief and loss. One participant described losing her sibling while pregnant and feeling the need to regulate her emotions to protect the pregnancy, “*I had to keep myself focused because I didn’t want to cause stress… and end up miscarrying my baby*” (P7).

#### 3.5.2 Extrinsic stressors

Employment was the most commonly described extrinsic stressor. One participant worried about job security during pregnancy due to lack of maternity leave protections, describing it as “*a huge like, ‘Oh my God, am I still going to have my job when this happens?*’” (P3). Another expressed concern about how a new employer might perceive a pregnant applicant (P1). Financial strain also emerged when family employment changed; one participant described her husband losing his job late in pregnancy, leaving her as the sole income earner and worrying about basic needs, “*There was some times of feeling like we did not have enough to make ends meet or that I was worried we would run out of food*” (P10).

Housing instability was another significant source of stress. One participant described experiencing homelessness during pregnancy and “*without having support from my family, my friends, and the father of my child*” (P4). Another reported being denied housing assistance despite completing the required steps and being unable to work due to pregnancy complications (P7). Transportation and distance also shaped access to care. Participants described traveling long distances for specialty care (P2) and relying on public transportation to attend appointments and access resources such as food pantries (P4).

Not all participants experienced substantial structural stressors. One described feeling “*super lucky*” to avoid transportation and financial barriers due to strong family support (P8). Overall, pregnancy often unfolded alongside broader social and economic challenges that shaped participants’ experiences of care and well-being.

### 3.6 Mental health

Participants described mental health during pregnancy and postpartum as shaped by emotional distress, prior loss, pregnancy complications, social stressors, and access to trusted support. Experiences ranged from minimal concerns to anxiety, depression, grief, and suicidal ideation.

#### 3.6.1 Mental health screening, stigma, and engagement with care

Experiences with mental health screening varied. Several participants recalled completing standardized questionnaires during prenatal or postpartum visits, often with limited follow-up. One participant explained that screening was “*just your standard PHQ-9*” without meaningful discussion (P3), while another felt postpartum screening was impersonal and would have preferred a direct conversation about how she was feeling. (P1).

Perceptions of screenings were mixed. Some viewed it as helpful for normalizing emotional changes, with one participant noting it helped providers understand “*what people are going through when they’re pregnant*” (P5). Others worried that screening outcomes could be stigmatizing. One participant reported intentionally minimizing symptoms to avoid being flagged, fearing differential treatment, “*as a mom, because I’m a Brown mom…*” (P8). She went on to explain that being labeled as having postpartum depression could increase the risk of outside intervention, particularly given broader assumptions about parenting among Native and other communities of color. “*It’s easy for People of Color and Native people to automatically be assumed to be poor parents… if you’re categorized as somebody who has postpartum depression, it’s a lot easier for someone to say that could be an unsafe home for a baby*” (P8).

Barriers to accessing behavioral health services were also described. One participant reported multiple unsuccessful attempts to access care through a tribal clinic, “*I had tried a few different times to get into our tribal health clinic. We have a behavioral health program there, and I was never able to get in for whatever reason; I never got a call back to set up an appointment*” (P10). At the same time, participants expressed interest in more relational and culturally responsive approaches, including conversations with trusted providers, peer support, and community-based models.

#### 3.6.2 Depression, anxiety, and emotional distress

Participants described a range of emotional responses during pregnancy and postpartum, including anxiety, depression, and feeling overwhelmed. Some linked emotional distress to pregnancy complications or physical strain. One participant explained that, “*I went through a lot of different things, so I was pretty depressed for a little bit of time there*” (P2).

Others described anxiety shaped by prior loss or trauma. One participant explained that later stages of pregnancy could trigger memories of a previous loss, noting that she experienced “*so much anxiety after week 25 because of that experience*” (P1). Some participants described postpartum depression, “*I have postpartum depression, and it got worse. It’s worse now. That’s why I have to be on medication*” (P4). Others described depressive symptoms after birth but chose to rely on family and friends rather than formal treatment.

In some cases, participants described more severe distress. One participant shared experiencing suicidal ideation during pregnancy, explaining, “*I didn’t have a plan, I just wanted everything to stop*” (P1); this led her to seek therapy. Another described managing pre-existing mental health conditions with medication during pregnancy and postpartum (P4).

### 3.7 Birth and postpartum experiences

#### 3.7.1 Delivery experiences

Delivery experiences among participants included vaginal births, scheduled cesarean deliveries, and emergency C-sections. While some described uncomplicated births, others experienced medically complex deliveries.

For some, complications during labor led to urgent surgical intervention. One participant explained that diabetes and preeclampsia contributed to an emergency C-section at 37 weeks and NICU admission for her baby (P7), though she later emphasized that her child was doing well. Another described being induced but requiring emergency surgery when her and her baby’s heart rates showed signs of distress (P5).

Participants highlighted the importance of supportive people during delivery. One participant described a cousin accompanying her in the operating room and singing traditional songs, which provided comfort during a frightening birth experience: “*She’s really traditional. She’s a Sun Dancer, and she knows how to sing… it was funny that she was ‘Lilili-ing’ in the [operating] room, and I was laying there*” (P5). She also emphasized that interactions with staff could shape birth experiences in lasting ways. Although many clinicians were supportive, a single negative interaction during her labor overshadowed the experience: “*it just takes that one person to ruin the whole thing*” (P5).

#### 3.7.2 Postpartum recovery and care

Postpartum experiences varied considerably. Some participants described routine follow-up and relatively smooth recovery, while others reported limited follow-up or feeling dismissed when complications arose. One participant reported experiencing infection symptoms after delivery and felt her concerns were dismissed until an emergency clinician treated her with antibiotics (P8).

Participants also described variability in the level of support provided during postpartum visits. One participant felt that care during the COVID-19 pandemic was less personal and more rushed compared with earlier pregnancies, noting, “*It was just get in and get out… ‘your incision looks great, we’ll see you in two weeks’… whereas before it was more personal*” (P1). Positive postpartum experiences were often linked to strong support systems. One participant described family and friends regularly checking on her well-being and helping with recovery: “*I had a lot of family and friends not only checking in on baby, but checking in on me… ‘Do you need anything? How are you doing?’*” (P6).

## 4 Discussion

This study centers Indigenous women’s pregnancy journeys as experiences shaped not only by healthcare encounters but also by relationships, cultural teachings, prior pregnancies, and broader social realities. Participants’ stories reflected strength, adaptation, and survivance through reliance on family networks, a desire for cultural knowledge, and self-advocacy within fragmented systems of care. This framing reinforces growing Indigenous maternal health scholarship which emphasizes pregnancy and birth as relational, cultural, and place-based experiences rather than solely focusing on disparities (6, 13, 41).

Although cultural teachings and traditions were important to participants, access was often inconsistent and rarely integrated into clinical care. Participants described wanting more culturally rooted guidance during their pregnancy journeys, consistent with prior work showing that Indigenous women want care that is not only clinically safe but also culturally grounded, relational, and reflective of Indigenous values and practices (6, 41). Participants’ efforts to seek, preserve, and reclaim cultural teachings during pregnancy also reflect Indigenous survivance, particularly in the context of healthcare systems historically shaped by colonial disruption and exclusion of Indigenous knowledge. Through the continuation and revitalization of cultural practices, storytelling, family involvement, and relational approaches to care, participants demonstrated survivance as an active expression of Indigenous presence, continuity, and self-determination within maternal health experiences (8, 10, 31). Culturally responsive maternity care, therefore, requires structural integration of Indigenous knowledge, ceremony, and family-centered approaches within perinatal systems.

Prior pregnancy experiences and fertility concerns shaped how women navigated subsequent pregnancies, influencing vigilance, emotional responses, and self-advocacy. These findings support a life course perspective in which pregnancy is not an isolated event, but part of an ongoing reproductive trajectory shaped by prior experiences and future expectations (36, 42).

Participants’ descriptions of prenatal care reflected both strengths and strains within current healthcare systems. Tribal clinics and IHS often served as important entry points and, in some cases, facilitated early identification of health risks, particularly for cardiometabolic conditions. However, consistent with national patterns, few participants had access to IHS or tribally operated facilities that provided full obstetric or birthing services, which are limited in number, necessitating referrals to external systems (25, 27, 43, 44). These transitions increased coordination burdens and created opportunities for delays and gaps in care, particularly in contexts shaped by geographic distance, referral complexity, and limited specialty availability (27, 28, 45, 46). At the same time, participants highlighted strengths of IHS care, including early and proactive screening practices, particularly for cardiometabolic conditions (47); underscoring the importance of tailoring care to population-specific risk profiles and supporting early intervention (48, 49). Overall, these findings illustrate how fragmented systems shift the burden of care coordination onto patients, reinforcing the need for improved integration and continuity across perinatal services.

Participants also described pregnancy care as shaped by mistrust, discrimination, and the enduring presence of historical trauma. Even when overt discrimination was not always named, women described entering healthcare settings with awareness of unequal treatment and poorer outcomes for Native patients. These findings are consistent with prior work showing that healthcare experiences for AIAN women are shaped by both contemporary inequities and histories of reproductive control, forced sterilization, child removal, and culturally unsafe care (6, 27, 42, 50, 51). These experiences further underscore the importance of trust-building and culturally clinical environments.

Cardiometabolic conditions were prominent in participants’ narratives and shaped pregnancy experiences, including anxiety (52), monitoring, and delivery decisions (49). Although this qualitative study was not designed to estimate prevalence, the frequency with which these conditions appeared in women’s narratives underscores how central cardiometabolic risk is to Indigenous pregnancy experiences in this sample (22, 53). This pattern is especially important given the well-documented role of hypertensive and cardiovascular conditions in severe maternal morbidity and pregnancy-related death (22).

Social support is consistently identified in the literature as a central protective factor in Indigenous maternal health, with family and community networks providing critical emotional, cultural, and practical support across the perinatal period (6, 10, 41, 51). At the same time, Indigenous women often navigate substantial structural stressors such as caregiving responsibilities, employment instability, food insecurity, housing challenges, intimate partner violence, transportation barriers, and grief, which are not often addressed within clinical care alone (54). Together, these findings reinforce the well-established role of social determinants of health in shaping maternal experiences and outcomes.

Persistent gaps in care, including limited access to culturally concordant providers, fragmented information, and a lack of structured peer support, have also been widely documented among Indigenous populations (45, 54). Importantly, participants did not simply describe wanting more services; they described wanting supports that were relational, consistent, culturally grounded, and able to bridge medical care with everyday realities, reinforcing previous scholarship showing that Indigenous maternal wellbeing is deeply relational and that support from family, peers, and community-based workers can buffer stress, improve navigation of care, and strengthen perinatal experiences (30, 32, 51). Community-based models such as community health workers, doulas, midwives, case managers, and home-visiting programs have demonstrated promise in addressing these gaps by improving care navigation, strengthening trust, and providing ongoing, culturally responsive support (10, 54–62).

Mental health was interwoven through participants’ experiences. While screening was common, it was often experienced as impersonal and insufficient, and some participants expressed concerns about stigma and surveillance. These experiences are important to consider in the context of recent CDC Maternal Mortality Review Committee data showing that mental health conditions are among the leading underlying causes of pregnancy-related death among AIAN women, and that 100% of pregnancy-related deaths in this population were determined to be preventable (63). The CDC also reports that most pregnancy-related deaths among AIAN women occur after delivery (83.7%), underscoring the importance of sustained mental health and postpartum support beyond the immediate perinatal period (63–65).

Prior research also suggests that standardized screening tools (e.g., PHQ-2, GAD-2) may miss a substantial proportion of individuals experiencing depression or anxiety, with one study showing detection rates of only approximately 57% among patients later diagnosed with these conditions (66). These limitations may be especially pronounced in settings where patients are reluctant to disclose symptoms due to stigma, mistrust, or fear of surveillance (64, 65, 67). In this context, participants’ experiences point to the potential value of more relational and culturally grounded approaches to mental health care, including ongoing conversations with trusted providers and community-based supports (64, 65, 67, 68).

Importantly, participants’ narratives reflected survivance alongside distress. Women described seeking therapy, relying on family, drawing from cultural teachings, advocating for themselves, and continuing to navigate pregnancy while carrying a substantial emotional burden. These findings suggest that strengthening perinatal mental health care for AIAN women may require approaches that both build on existing strengths and address gaps in relational, culturally responsive, and continuous support. One such approach, cultural safety, has been conceptualized by Indigenous midwives beyond the United States. Culturally safe practices around birthing encompass provider-patient relationships, integrated knowledge of Indigenous and Western birthing protocols, and physical spaces that reflect Indigenous values (69).

Postpartum experiences highlighted gaps in continuity and responsiveness of care, with variability in follow-up and instances in which concerns were dismissed or inadequately addressed. These findings align with broader evidence describing a “postpartum cliff,” (70) in which care is limited, brief, and disconnected from ongoing recovery needs, stressing the need to redesign postpartum care (71, 72).

An Indigenous lens suggests a model of postpartum care grounded in continuity, relationality, and community support. Improving postpartum care will require systems that extend beyond episodic clinical surveillance to incorporate Indigenous ways of caring for mothers and babies, strengthen care transitions, integrate behavioral health and social supports, and treat postpartum recovery as an ongoing relational process rather than a single follow-up encounter (71, 72).

Future research should focus on developing and rigorously evaluating interventions that bridge clinical care with community-based and culturally grounded supports, particularly those that enhance continuity, trust, and navigation across systems. Research is also needed to identify scalable models that can be adapted across diverse tribal and urban Indigenous contexts while maintaining cultural relevance and community leadership. The SAFE (Securing AIAN Futures through eHealth) intervention, informed by the findings of this study, represents one approach to addressing these identified gaps through integration of community health workers, culturally responsive engagement, and remote support. Continued work is needed to test and refine such models and to advance broader system-level changes that honor Indigenous knowledge, strengthen community capacity, and improve maternal and infant outcomes.

### 4.1 Recommendations

Findings from this study highlight the need for multi-level strategies to address gaps in communication, care coordination, and cultural responsiveness across perinatal and postpartum care systems. We offer the following recommendations to advance more equitable and culturally responsive care, spanning clinical practice, health systems, and policy-level interventions:

- Increase integration of CHWs, doulas, midwives, and Indigenous birth workers to support care navigation, patient advocacy, and culturally grounded education for patients and providers.
- Support provider training and system-level changes that allow for culturally informed care, including patient-led birth planning and the integration of Indigenous cultural practices and ceremonies.
- Develop models that enhance communication across providers and systems, drawing on relationship-based and continuity-focused approaches similar to chronic disease management.
- Move beyond standardized screening tools (e.g., mental health, intimate partner violence, substance use, etc.) toward relational, culturally responsive approaches that prioritize trust, dialog, and meaningful follow-up.
- Extend support beyond delivery to address ongoing mental health and physical health needs during the postpartum period.
- Invest in solutions targeting transportation, food insecurity, and access to maternity and specialty care, alongside early identification and management of cardiometabolic risk.

### 4.2 Limitations

This study has several limitations. The sample size was small and not intended to be representative; rather, the goal was to capture in-depth experiences of pregnancy. Recruitment challenges, including the use of social media platforms, introduced limitations, such as bot responses, resulting in a lower-than-expected ratio of eligible participants to those expressing interest in the study. Additionally, most participants’ experiences reflected connections to urban settings and may not fully represent those living in more rural or tribally governed contexts. Despite these limitations, this study provides important insights into the lived experiences of AIAN women navigating pregnancy across complex healthcare and social systems.

## 5 Conclusion

This study highlights the strength, adaptability, and survivance that Indigenous women bring to their pregnancy journeys, even as they navigate fragmented healthcare systems, limited cultural integration, and gaps in sustained support across the perinatal period. Participants’ experiences underscore that improving maternal health for AIAN communities requires more than expanding services. It requires reorienting care toward relational, culturally grounded, and continuous models across pregnancy and postpartum. These findings point to the importance of approaches that reduce system fragmentation, strengthen care coordination, and center trusted relationships through supports such as community health workers, doulas, midwives, and Indigenous birth workers. Integrating cultural knowledge, community connection, and patient advocacy into perinatal care is essential to advancing both equity and quality in maternal health.

## Data Availability

The datasets presented in this article are not readily available because due to the sensitive nature of qualitative data and the risk of participant re-identification, full datasets are not publicly available. De-identified data may be made available to qualified researchers upon reasonable request, subject to Institutional Review Board (IRB) approval, data use agreements, and review and approval by the study’s Indigenous Publications and Dissemination Committee, in alignment with protections for participant confidentiality and Indigenous data sovereignty principles. Requests to access the datasets should be directed to Christina Pacheco, cpacheco@kumc.edu or Taneisha Scheuermann, TScheuermann@kumc.edu.

## Author’s note

Consistent with best practices in the field of Indigenous studies 66, we use the terms American Indian and Alaska Native (AIAN) to refer to our sample and to specify information specific to Indigenous peoples of North America. We use the term Indigenous when referring to academic scholarship (e.g., examining through an Indigenous lens) and when referring to or including people groups outside of North America.
