# Supplementary material for: Pregnancy as a journey of care and connection: Indigenous women’s experiences navigating health systems and community support
Source: Front Public Health. 2026 Jun 9;14:1839105. doi: 10.3389/fpubh.2026.1839105 (PMC13286933; doi:10.3389/fpubh.2026.1839105)
Supplement: Supplementary file 1 [file Supplementary_file_1.docx]

**Interview Guide: Pregnancy Journey Mapping for Pregnant and Postpartum Women**

The purpose of this interview is to better understand how pregnant and postpartum Native women navigate pregnancy, healthcare systems, cultural practices, and community resources. We are interested in learning about experiences, supports, barriers, and opportunities to improve care and services for women and families.

- ***Do Journey Map***
- Initial Pregnancy Experience: Describe your experience and actions when you first realized you may be pregnant.
  - What questions and feelings came up for you?
  - What were your next steps?
  - Who did you turn to for these questions?
- When did you first engage with a health care provider (doctor, physician assistant, nurse)?
  - Did you engage a traditional healer?
    - Describe your experience with these providers.
  - What tribal or familial rules, guidelines, and stories surround pregnancy in your culture? Did you discuss these with your medical providers?
  - Did you utilize other social services (food pantry, social worker, rental assistance, etc.) and/or a community health worker to support your journey? Describe that experience.
    - How did these services impact your pregnancy journey?
- Supports:
  - What supports were missing from your pregnancy journey?
  - What supports did you receive that were impactful (positive & negative)to your experience?
- Did you receive culturally responsive care from your providers?
  - If not, what could have been done better?
  - If so, what was done well?

*Identify the most significant social determinants of health, and collect via survey (what things, behaviors, environments could have impacted your health or the health of your baby during your pregnancy?)*
